# Association of Promoter Methylation of *VGF* and *PGP9.5* with Ovarian Cancer Progression

**DOI:** 10.1371/journal.pone.0070878

**Published:** 2013-09-27

**Authors:** Mariana Brait, Leonel Maldonado, Maartje Noordhuis, Shahnaz Begum, Myriam Loyo, Maria Luana Poeta, Alvaro Barbosa, Vito M. Fazio, Roberto Angioli, Carla Rabitti, Luigi Marchionni, Pauline de Graeff, Ate G. J. van der Zee, G. Bea A. Wisman, David Sidransky, Mohammad O. Hoque

**Affiliations:** 1 Department of Otolaryngology – Head and Neck Surgery, Johns Hopkins University School of Medicine, Baltimore, Maryland, United States of America; 2 Clinical Research Coordination, Instituto Nacional de Câncer (INCA)-Brazilian National Cancer Institute, Rio de Janeiro, Brazil; 3 Department of Gynecologic Oncology, University Medical Center Groningen, University of Groningen, Groningen, The Netherlands; 4 Department of Pathology, Johns Hopkins Medical Institutions, Baltimore, Maryland, United States of America; 5 Department of Biosciences, Biotechnologies and Biopharmaceutics, University of Bari, Bari, Italy; 6 Department of Pathology, Hospital San Jose Tec de Monterrey, Monterrey, Nuevo Leon, Mexico; 7 Laboratory for Molecular Medicine and Biotechnology Center for Integrated Research, University Campus Bio-Medico of Rome, Rome, Italy; 8 Department of Gynecology, University Campus Bio-Medico of Rome, Rome, Italy; 9 Department of Pathology, University Campus Bio-Medico of Rome, Rome, Italy; 10 Department of Oncology, Johns Hopkins University School of Medicine, Baltimore, Maryland, United States of America; 11 Department of Urology, Johns Hopkins University School of Medicine, Baltimore, Maryland, United States of America; The Chinese University of Hong Kong, Hong Kong

## Abstract

**Purpose:**

To elucidate the role of biological and clinical impact of aberrant promoter hypermethylation (PH) in ovarian cancer (OC).

**Experimental Design:**

PH of *PGP9.5*, *HIC1*, *AIM1*, *APC*, *PAK3*, *MGMT*, *KIF1A*, *CCNA1*, *ESR1*, *SSBP2, GSTP1, FKBP4* and *VGF* were assessed by quantitative methylation specific PCR (QMSP) in a training set. We selected two genes (*VGF* and *PGP9.5*) for further QMSP analysis in a larger independent validation (IV) set with available clinical data. Biologic relevance of *VGF* gene was also evaluated.

**Results:**

PH frequency for *PGP9.5* and *VGF* were 85% (316/372) and 43% (158/366) respectively in the IV set of samples while no PH was observed in controls. In 372 OC cases with available follow up, *PGP9.5* and *VGF* PH were correlated with better patient survival [Hazard Ratios (HR) for overall survival (OS) were 0.59 (95% Confidence Intervals (CI)  = 0.42–0.84, p = 0.004), and 0.73 (95%CI = 0.55–0.97, p = 0.028) respectively, and for disease specific survival (DSS) were 0.57 (95%CI 0.39–0.82, p = 0.003) and 0.72 (95%CI 0.54–0.96, p = 0.027). In multivariate analysis, *VGF* PH remained an independent prognostic factor for OS (HR 0.61, 95%CI 0.43–0.86, p<0.005) and DSS (HR 0.58, 95%CI 0.41–0.83, p<0.003). Furthermore, *PGP9.5* PH was significantly correlated with lower grade, early stage tumors, and with absence of residual disease. Forced expression of *VGF* in OC cell lines inhibited cell growth.

**Conclusions:**

Our results indicate that *VGF* and *PGP9.5* PH are potential biomarkers for ovarian carcinoma. Confirmatory cohorts with longitudinal follow-up are required in future studies to define the clinical impact of *VGF* and *PGP9.5* PH before clinical application.

## Introduction

Ovarian cancer (OC) is the second most common gynecological cancer and the leading cause of death among gynecological cancers worldwide [1]. 22,240 new cases were estimated for 2013 in the United States, leading to 14,030 deaths from this cancer type [2]. The median age of patients with OC is 60 years, and the average lifetime risk for developing OC in women is about 1 in 70 [3]. Seventy percent of patients with OC have advanced disease (stage III or IV) at the time of diagnosis, with a 5-year survival rate of 15 to 20% despite aggressive treatment [4], in comparison to the early stage patients with a survival rate above 90% [3]. OC has been generally treated with platinum-based chemotherapy and often recurs due to acquired platinum resistance. The initial clinical response to platinum-based drugs is a major determinant of outcome for patients with OC. Patients with tumors demonstrating in vitro extreme drug resistance to platinum were found to be at a significantly increased risk for progression and death when treated with standard platinum-based regimens [5]. It is therefore of major significance to identify useful predictive markers indicating platinum sensitivity. These may allow better treatment selection for the 1^st^ line of treatment, possibly allowing better outcome for the platinum resistant patients. Determination of appropriate markers to anticipate response to standard chemotherapeutic or newer biologic agents will allow for improved control and cure rates for OC, as well as selection of adjuvant therapy or identification of patients appropriate for specific clinical trials. Furthermore, the ability to predict OC outcomes after surgical resection is critical for clinicians, as it would impact the use of adjuvant chemotherapy and radiation therapies. An independent prognostic indicator of OC survival would therefore be invaluable to physicians and patients in selecting treatment options.

It is known that genetic (changes in DNA sequence such as deletions, amplifications and mutations) and epigenetic changes (defined as heritable changes in gene expression that occur without changes to the DNA sequence) contribute to the development and progression of tumor cells [6]. The most common epigenetic events include DNA methylation and histone acetylation [7], being DNA methylation possibly the most widely studied aspect of epigenetics with regard to carcinogenesis, and the key focus of pharmacologic interventions in clinical trials. It refers to the addition of a methyl group to the 5-carbon position of the cytosine ring to form 5-methylcytosine (5mC), but only on cytosines that precede a guanosine in the DNA sequence, known as CpG dinucleotide [8]. There are CpG-rich regions known as CpG islands which usually span the 5′end region (promoter, untranslated region and exon 1) of many genes with tumor suppressor activity and are usually unmethylated in normal cells [9]. The human genome in this normal cells is not methylated uniformly, containing unmethylated segments interspersed with methylated regions [10], whereas cancer cells methylation patterns are altered, undergoing global DNA hypomethylation [11], as well as hypermethylation of certain CpG islands [8]. Aberrant CpG island hypermethylation (in particular in tumor suppressor genes' promoter) as well as histone modification lead to transcriptional inactivation and gene silencing, being a common phenomenon in human cancer cells and likely one of the earliest events in carcinogenesis [7]. In particular, promoter hypermethylation (PH) is a frequent mechanism of inactivation of tumor suppressor genes (TSGs) [7], [8] and PH of certain cancer related genes were found to be associated with therapeutic response and outcome of the disease [12], [13].

In the present study, we analyzed PH of 13 genes *(PGP9.5*, *HIC1*, *AIM1*, *APC*, *PAK3*, *MGMT*, *KIF1A*, *CCNA1*, *ESR1*, *SSBP2, GSTP1*, *VGF, FKBP4)* by quantitative fluorogenic real-time methylation specific PCR (QMSP) in a training set and finally tested two genes (*VGF* and *PGP9.5*) in an independent validation set to evaluate whether there is any association of PH of these two genes with any clinical factors including overall outcome of the patient. Moreover, we studied the functional role of VGF as an anti-tumorigenic molecule in OC derived cell lines.

## Materials and Methods

### Study cohort

#### Training Set

In order to compose the first set of samples we obtained samples of ovarian tumor tissue from our tissue archive, collected from 33 patients with epithelial OC who underwent therapeutic surgery at The Johns Hopkins University School of Medicine. We also obtained samples from 24 patients with OC who underwent surgery at University Campus Bio-Medico, Rome Italy. To be included in the cohort, an eligible patient had to have a confirmed diagnosis of OC and a sufficient amount of archived tumor material for DNA extraction. The samples were preserved in paraffin, and a set of slides (10 microns of thickness) were taken from each block. The demographic and clinical information was obtained from the computerized tumor registry at The Johns Hopkins Healthcare System or from the registry at Department of Obstetrics and Gynecology, University Campus Bio-Medico of Rome, Italy. The tumors were classified according to the FIGO staging system [14]. From the Johns Hopkins patients (33), 16 patients presented with early stage disease (15 stage I and 1 stage II) and 17 with advanced disease (14 stage III and 3 stage IV). All the samples were epithelial ovarian carcinomas with a serous-papillary histology. From the 24 patients from Italy, 10 had early stage disease (6 with stage I, and 4 with stage II), 13 had advanced disease (12 with stage III, and 1 with stage IV) and 1 had unknown stage. Eighteen patients presented with epithelial ovarian carcinomas (serous, endometrioid, mucinous, squamous, undifferentiated), one with germ cell tumor, four presented with secondary (metastatic) tumors, and 1 patient had unknown histology. The normal ovarian epithelium tissue was obtained from 13 patients who underwent prophylactic surgery for any benign condition in Hospital San Jose Tec de Monterrey in Mexico. A detailed summary of the training set of patients is available in **Table 1(A)**.

**Table 1:** Demographic and clinicopathological data of Ovarian Cancer samples (Training set and independent validation set) and Normal Ovarian samples.

				
	1st portion of Cases (n = 33)	2nd portion of cases (n = 24)	Training set (Total)	Controls(n = 13)
**Age at diagnosis**				
Median	57	56	57	46
Range	23–79	37–77	23–77	40–55
**Follow-up (months**)				
Median	24	37	30.5	
Range	0–228	13–79	0–228	
**Race**				
Caucasian	24	24	48	0
African-american	8	0	8	0
Hispanic	0	0	0	13
Unknown	1	0	1	0
**Tumor type**				
EOC*	33	18	51	
Germ cell tumor	0	1	1	
Metastasis	0	4	4	
Unknown	0	1	1	
**Histology**				
Serous-papillary	33	11	44	
Endometrioid	0	4	4	
Mucinous	0	2	2	
Undifferentiated	0	1	1	
Unknown	0	6	6	
**Stage**				
I	15	6	21	
II	1	4	5	
III	14	12	26	
IV	3	1	4	
Unknown	0	1	1	
**Grade**				
Borderline	12	0	12	
G1	0	2	2	
G2	8	5	13	
G3	13	12	25	
GX**	0	1	1	
Unknown	0	4	4	
**Chemotherapy**				
Yes	11	23	34	
No	9	0	9	
Unknown	13	1	14	
**Type of chemotherapy**				
Platinum/taxol after surgery	0	18	18	
Platinum/taxol before and after surgery	0	6	6	
Unknown	33	0	33	
**Recurrence**				
Yes	4	14	18	
No	0	10	10	
Unknown	29	0	29	
**Metastasis**				
Yes	1	0	1	
No	32	0	32	
Unknown	0	24	24	
**Smoking**				
Smoker	2	5	7	
Non-smoker	16	19	35	
Unknown	15	0	15	
**Alcohol**				
Current	6	2	8	
No	12	22	34	
Unknown	15	0	15	
**B. Validation set**				
**Ovarian cancer patients n = 372**				
**Age at diagnosis**				
Median	61			
Range	21–89			
**Follow-up time (months**)				
Median	30			
Range	0–234			
Unknown n = 1				
**Histology**	n	%		
Serous	227	61%		
Mucinous	43	12%		
Endometroid	39	10%		
Clear cell	18	5%		
Adenocarcinoma	13	3%		
Other	31	8%		
Unknown	1	0%		
**Grade**				
Borderline	5	1%		
I	57	15%		
II	101	27%		
III	154	41%		
Undifferentiated	19	5%		
Unknown	36	10%		
**FIGO Stage**				
I	67	18%		
II	30	8%		
III	224	60%		
IV	51	14%		
**Residual disease after surgery**				
<2 cm	184	49%		
>2 cm	160	43%		
Unknown	28	8%		
**Chemotherapy**				
No chemotherapy	56	15%		
Platinum-containing	173	47%		
Platinum and taxane containing	105	28%		
Other	30	8%		
Unknown	8	2%		
	316			
**Borderline tumors n = 17**				
**Age at diagnosis**				
Median	50			
Range	19–77			
*Unknown (n = 5)*				
**Cystadenoma n = 18**				
**Age at diagnosis**				
Median	30			
Range	26–55			
*Unknown (n = 15)*				

* Epithelial ovarian cancer ^**^ Grade cannot be assessed.

#### Independent Validation (IV) Set

We analyzed an independent validation set for the most promising genes. This independent set consisted of 372 ovarian tumors, 17 borderline tumors and 18 ovarian cystadenomas (all preserved as fresh frozen samples). These patients underwent surgery at the University Medical Center Groningen, Groningen (UMCG), The Netherlands. The demographic and clinical information was obtained from the registry at the Department of Gynecologic Oncology, UMCG, The Netherlands. A detailed summary of the demographic and clinicopathological parameters of these samples is shown in **Table 1(B)**.

Approval for research on human subjects was obtained from The Johns Hopkins University institutional review boards. This study qualified for exemption under the U.S. Department of Health and Human Services policy for protection of human subjects [45 CFR 46.101(b)]. IRB guidelines were followed at each of the involved institutions. All human materials used in the study from the Department of Obstetrics and Gynecology, University Campus Bio-Medico of Rome, Italy, were collected according to the guidelines of the Local Ethical Committee of Campus Bio-Medico of Rome. The guidelines of the Local Ethics Committee, which are in default compliant to the Italian Protection Data Authority, determines that samples collected retrospectively from the Department of Pathology (formalin-fixed, paraffin-embedded tissue) don't need to have any approval and are exempted from obtaining written informed consent. According to the Italian Protection Data Authority rules (http://www.garanteprivacy.it/web/guest/home_en doc web n 1884019), Italian Institutions are in default authorized to use the samples and the corresponding clinical data included in the study. All samples collected from Hospital San Jose Tec de Monterrey, Monterrey, Mexico were collected following the Ethical Committee rules. According to the guidelines from the Mexican law for Health Research (Reglamento de la Ley General de Salud en Materia de Investigación para la Salud, article 23^rd^, 2^nd^ title, 1^st^ chapter: retrospective studies are considered as non-risk studies and therefore do not require a written informed consent of the participants or Ethical Committee approval. Samples from Mexico were from archived samples (formalin-fixed, paraffin-embedded tissue) and were retrieved from the hospitals' pathology archive. For the patients obtained from the UMCG in The Netherlands, patients gave informed consent for collection and storage of tissue samples in a tissue bank for future research. All relevant patient data were retrieved and transferred into an anonymous, password-protected, database. The patients' identity was protected by study-specific, unique patient codes and their true identity was only known to two dedicated data managers. According to Dutch regulations, these precautions meant no further institutional review board approval was needed (http://www.federa.org/). All patients' data were de-identified for the researchers in all 4 institutions.

### Gene Selection

In the present study, we selected a total of 13 genes to analyze their methylation status (using QMSP) by a candidate gene approach. Genes were selected based on their cancer specific methylation in OC and/or any other solid cancer types. The genes selected were: *PGP9.5*, *HIC1*, *AIM1*, *APC*, *PAK3*, *MGMT*, *KIF1A*, *CCNA1*, *ESR1*, *SSBP2, GSTP1, VGF* and *FKBP4*. A detailed summary of information about these genes is available in **Table S1 in File S1.**

Based on the alterations of above mentioned genes in cancer (further discussed in the discussion section), we analyzed all these 13 genes in a training set of samples and selected two genes (*VGF* and *PGP9.5*) for further analysis of a large well annotated independent set of samples. The selection criteria of the genes to be tested in the independent validation set were: a) Methylation frequency in tumor samples in the training set; b) Methylation frequency in normal samples; C) Novelty of the gene for OC; D) Known functions of each gene by literature/PubMed search.

### DNA extraction

After initial patient de-identification, all original OC histologic slides were reviewed to reconfirm the diagnosis by a senior pathologist. A representative block was retrieved for DNA extraction. Histologic slides from the formalin-fixed, paraffin-embedded tissue were taken. Slides were microdissected to obtain >70% neoplastic cells. The microdissected normal ovarian epithelium was isolated from ovarian tissue resected during surgery, confirming afterwards the absence of any malignant and/or pre-malignant process in the tissue. DNA was extracted using the phenol-choloroform extraction protocol followed by ethanol precipitation, as described previously [15]. For the UMCG specimens, DNA was isolated using standard salt-chloroform extraction and isopropanol precipitation. Precipitated DNA was resuspended in Tris-EDTA buffer (10 mM Tris; 1 mM EDTA, pH 8.0). Genomic DNA was amplified in a multiplex PCR according to the BIOMED-2 protocol, to check the DNA quality [16].

### Sodium Bisulfite Treatment

DNA extracted from primary tumors and normal ovarian epithelium was subjected to sodium bisulfite treatment, which converts unmethylated cytosine residues to uracil residues, as described previously [17]. For this, the EpiTect Bisulfite kit (Cat No. 59104, QIAGEN Inc., Valencia, CA) was used as per the manufacturer instructions. For the UMCG samples, bisulfite treatment was performed with the EZ DNA methylation kit according to manufacturer's protocol (Zymogen, BaseClear, Leiden, the Netherlands). After treatment, DNA was stored at −80°C until used.

### Methylation Analysis

Bisulfite-treated DNA was used as a template for fluorescence-based real-time PCR, as previously described [17]. Amplification reactions were carried out in triplicate in a final volume of 20 μL that contained 3 μL of bisulfite-modified DNA; 600 nM concentrations of forward and reverse primers; 200 nM probe; 0.6 U of platinum Taq polymerase (Invitrogen, Frederick, MD); 200 μM concentrations each of dATP, dCTP, dGTP and dTTP; and 6.7 mM MgCl_2_. Primers and probes were designed to specifically amplify the promoters of the 13 genes of interest and the promoter of a reference gene, *ACTB*; primer and probe sequences and annealing temperatures are provided in **Table S2 in File S1.** Amplifications were carried out using the following profile: 95°C for 3 minutes, followed by 50 cycles at 95°C for 15 seconds and 60°C for 1 minute. Amplification reactions were carried out in 384-well plates in a 7900HT sequence detector (Perkin-Elmer Applied Biosystems, Beverly, MA) and were analyzed by a sequence detector system (SDS 2.4; Applied Biosystems). Each plate included patient DNA samples, positive (*in vitro* methylated leukocyte DNA) and negative (normal leukocyte DNA or DNA from a known unmethylated cell line) controls, and multiple water blanks. Leukocyte DNA from a healthy individual was methylated *in vitro* with excess SssI methyltransferase (New England Biolabs Inc., Beverly, MA) to generate completely methylated DNA, and serial dilutions (90–0.009 ng) of this DNA were used to construct a calibration curve for each plate. All samples were within the assay's range of sensitivity and reproducibility based on amplification of internal reference standard (threshold cycle [CT] value for *ACTB* of 40). The relative level of methylated DNA for each gene in each sample was determined as a ratio of methylation specific PCR-amplified gene to *ACTB* (reference gene) and then multiplied by 1000 for easier tabulation (average value of triplicates of gene of interest divided by the [average value of triplicates of *ACTB*] ×1000). The samples were categorized as unmethylated or methylated based on the sensitivity of the assay.

For *VGF*, bisulfite sequence analysis was also performed to confirm the methylation status in the cell lines derived from ovarian cancer (IGROV, IGROV CP, A2780, A2780 CP, 2008 and 2008 C13) and from normal ovarian surface epithelium (OSE) cell lines (OSE2A, OSE2B and OSE7). Bisulfite treated DNA was amplified for the 5′ region that included at least a portion of the CpG island within 1 kb of the proposed transcription start site (TSS), contemplating the same area as the QMSP primers and probe. The primers were designed not to contain CpG sites, and considering the bisulfite treated sequence, in order to amplify the region regardless of its methylation status, allowing us to observe it without a reaction bias. The primers sequence were: Forward 5′-TTTGTTTTTGTTAGGGGGTTGTT-3′ and Reverse 5′- AACACCAATAAAAACTAATACTA-3′. PCR products were gel purified using the QIAquick Gel Extraction kit (Qiagen, Valencia, CA) according to the manufacturer's instructions. Each amplified DNA sample was sequenced by the Applied Biosystems 3700 DNA analyzer using forward or reverse primers and BD terminator dye (Applied Biosystems, Foster City, CA). Three independent reactions were run to confirm the observed data.

### Statistical Analysis

Statistical analysis was performed with IBM SPSS Statistics 19.0 (SPSS Inc., Chicago, IL). For each gene a methylation threshold (cut-off) above the highest value in the control group was used to achieve 100% specificity in the training set and the same cut-off values were applied for *VGF* and *PGP9.5* in independent validation set, analysis were also performed using a cutoff in the 75^th^ percentile of methylation values. Differences in methylation proportion between normals and cancers were assessed by Fisher's exact test. Furthermore, hypermethylation values for each gene were also compared between cancer and normal by Mann-Whitney U test. Correlations of the methylation levels of genes were assessed with Spearman correlation coefficients. The association between hypermethylation and the clinico-pathological characteristics was assessed by logistic regression, chi-square and Fisher's exact tests, as appropriate. Overall survival (OS) was defined as the time from diagnosis to death of any cause or last follow-up visit alive. Disease-specific survival (DSS) was defined as the period from diagnosis to death as a consequence of OC. Differences in OS and DSS according to clinicopathological characteristics and methylation of genes were analyzed using Cox regression analysis. All significant variables with a *P value* <0.05 in univariate analysis were included in multivariate analysis. *P value*s of <0.05 were considered statistically significant. All the *P values* are two-sided.

### 5-aza-2′-deoxycytidine treatment of cells and Reverse transcription-PCR and real-time reverse transcription-PCR

We seeded (density: 1×10^6^) six ovarian cancer cell lines (IGROV, IGROV CP, A2780, A2780 CP, 2008 and 2008 C13) lines in their respective culture medium and maintained them for 24 h before treating them with 5 µM 5-aza-2′-deoxycytidine (5-aza-dC; Sigma) for 5 to 7 days. We renewed medium containing 5-aza-dC every 24 h during the treatment. We handled the control cells (mock treated) in the same way, without adding 5-aza-dC. We also treated 5-aza-dC in combination with trichostatin A (TSA, Sigma, St. Louis, MO), a histone deacecetylase inhibitor, and TSA alone. Stock solutions of 5-aza-dC were dissolved in phosphate buffer saline PBS (pH 7.5). We prepared total RNA using Qiazol (Qiagen Inc. Valencia, CA), following manufacturer's instructions.

### Reverse transcription-PCR (RT-PCR)

cDNA was synthetized using Superscript III (Invitrogen, Life Technologies, Carlsbad, CA), with an initial amount of 1ug of RNA, following its standard protocol. RT-PCR was performed as described previously [18]. One microliter of each cDNA was used for real-time RT-PCR using specific primers and taqman probe for the gene of interest. Amplifications were carried out in 384-well plates in a 7900HT Sequence Detector System (Applied Biosystems, Foster City, CA). Expression of genes relative to glyceraldehyde-3-phosphate dehydrogenase (GAPDH) was calculated based on the threshold cycle (Ct) as 2^−Δ(ΔCt)^, where ΔC_t_  =  C_t,GENE_−C_t,GAPDH_ and Δ (ΔC_t_)  =  ΔC_t,AZA_−ΔC_t,M_ (M, mock treatment; Aza, 5-aza-dC treatment) [19].

### Colony focus assay

We compared the methylation profile and expression of *VGF* in 3 normal ovarian surface epithelium (OSE) cell lines (OSE2A, OSE2B and OSE7) and 3 pairs of isogenic OC cell lines (A2780 and A2780CP, 2008 and 2008C13, and IGROV and IGROV CP) different in regards to cisplatin sensitivity. We then performed focus formation assay for VGF using two cell lines (2008 and 2008C13) that were methylated and not expressing *VGF.*

OC cell lines 2008 and 2008C13 were seeded in 10 cm dishes and transfected with VGF expression vector (pCMV6-AC-VGF-GFP) and empty vector (pCMV6-AC-GFP) (Origene, Rockville, MD). Twenty-four hours after transfection, cells were split into several dishes. From the following day, cells were cultured for 2 weeks in medium containing 400 µg/mL and 30 µg/mL of G418 (Cellgro, Manassas, VA) for 2008 and 2008C13 respectively. After 2 weeks cells were washed twice with PBS, fixed with 25% acetic acid and 75% methanol at room temperature for 10 minutes and then stained with 0.1% crystal violet. Colonies were counted and the number of colonies per dish was averaged from three independent experiments (colonies >2 mm in diameter were considered as positive). To confirm the expression of VGF, cells were harvested 48 hours after transfection and western blot analysis was performed, utilizing VGF antibody (Santa Cruz, CA), normalized by β-actin levels (antibody from Sigma-Aldrich, Saint Louis, MO).

## Results

### Overall methylation frequency on OC Samples and Normal Ovarian Samples (Training Set)

Thirteen genes (*PGP9.5*, *HIC1*, *AIM1*, *APC*, *PAK3*, *KIF1A*, *CCNA1*, *ESR1*, *SSBP2, GSTP1*, *MGMT, VGF* and *FKBP4*) were analyzed for PH using QMSP in two different set of samples. The observed methylation frequencies of each gene for the training set are summarized in **Table 2A**. Briefly, in the training set (total), the most frequently methylated genes were: *PGP9.5*, *HIC1*, *ESR1 and VGF*. The observed frequency of methylation in this set was 19% (11/57) for *ESR1*, 67% (38/57) for *HIC1*, 28% (16/57) for *PGP9.5* and 37% (21/57) for *VGF*. Each of the latter 4 genes were significantly more methylated in OC than normal (p = <0.05 for each gene). Combining all 4 genes analyzed in the training set, we observe methylation in at least 1 of the 4 genes in 81% (46/57) of the OC samples, with a specificity of 100%.

**Table 2:** Promoter Methylation Frequencies of Candidate genes in Gene Evaluation Sets and Validation Set.

A. Promoter methylation frequency for the 13 genes analyzed in the evaluation set of ovarian cancer samples and 13 normals.										
1^st^ Set 1 of samples				Additional cancer cases			Training Set (Total)			
	Methylation positive % (number of methylation positive/number of total cases)			Methylation positive % (number of methylation positive/number of total cases)			Methylation positive % (number of methylation positive/number of total cases)			Cut-off*
GENE	TUMOR	NORMAL	*P* value	TUMOR		*P* value	TUMOR		*P* value	
***ESR1***	18 (6/33)	0/13	0.163	21 (5/24)		0.140	19 (11/57)		0.031	>59.50
***HIC1***	61 (20/33)	0/13	<0.001	75 (18/24)		<0.001	67 (38/57)		0.000	>270.70
***PGP9.5***	24 (8/33)	0/13	0.084	33 (8/24)		0.032	28 (16/57)		0.031	>0
***VGF***	24 (8/33)	0/13	0.084	54 (13/24)		0.001	37 (21/57)		0.007	>0
***AIM1***	3 (1/33)	0/13	1.000							>0
***APC***	15 (5/33)	0/13	0.301							>0
***CCNA1***	3 (1/33)	0/13	1.000							>0
***FKBP4***	9 (3/33)	0/13	0.548							>0
***GSTP1***	0 (0/33)	0/13	ND			N.D				>4.34
***KIF1A***	6 (2/33)	0/13	1.000							>2.35
***MGMT***	3 (1/33)	0/13	1.000							>9.75
***PAK3***	3 (1/33)	0/13	1.000							>242.86
***SSBP2***	9 (3/33)	0/13	0.548							>26.08

B. Promoter methylation frequency for the two genes analyzed in the validation set of ovarian cancer samples										
	Methylation positive % (number of methylation positive/number of total cases)									
GENE	Tumors	Borderline tumors	*Cystadenomas*							
*PGP9.5*	85% (316/372)	100% (18/18)	71% (12/17)							
*VGF*	43% (158/366)	33% (6/18)	31% (5/16)							

* **Cut-off above highest control, so specificity is set on 100%**.

This set of tumor samples were analyzed for gene pre-selection purpose only. To maximize specificity, an empiric cut-off value was determined above the highest value in the control group for each gene. The cut-off value of each gene is shown in **Table 2A**.

### Association between DNA methylation changes and clinicopathological factors in the training set

We compared the methylation status of two genes (*PGP9.5* and *VGF*) in 57 OC samples of the training set to demographic and clinicopathological parameters such as age, stage, histological type, grade, smoking and alcohol consumption history of the patients, by logistic regression analysis. *VGF* methylation was more frequently present in earlier stages (Stage I and II) of OC in comparison with late stage (Stage III and IV) of tumors [14/26 (54%) versus 6/30 (20%) methylated] (Odds ratio, O.R = 0.21, 95C.I. [0.07–0.7], *p* value  = 0.011). Correlations with all other clinicopathologic parameters were not significant with methylation of any of the other genes (**Table 3A**).

**Table 3:** Correlation of demographic and pathologic patients' characteristics with gene-specific promoter methylation.

A. Training Set										
	*PGP9.5*					*VGF*				
Variables										
	P value		OR	95% C.I.		P value		OR	95% C.I.	
Age (continuous)	0.372		1.02	0.98	1.07	0.738		0.99	0.95	1.03
Stage (III, IV)	0.132		0.40	0.12	1.32	**0.011**		**0.21**	**0.07**	**0.70**
Histology (serous-pappilary)	0.099		0.25	0.05	1.30	0.206		0.35	0.07	1.78
Grade (II, III)	0.123		0.36	0.10	1.32	0.163		0.41	0.12	1.44
Smoking (yes)	0.316		0.32	0.03	2.97	0.408		2.00	0.39	10.34
Alcohol (yes)	0.657		1.44	0.29	7.21	0.651		1.43	0.30	6.70

B. Independent Validation Set									
	*PGP9.5*					*VGF*			
Variables									
	P value		OR	95% C.I.		P value	OR	95% C.I.	
Age (>61)	0.230		0.75	0.47	1.20	0.927	0.98	0.65	1.48
Histology (serous)	**<0.001**		**0.24**	**0.14**	**0.40**	0.460	0.84	0.53	1.33
Grade (III/undifferentiated)	**0.012**		**0.52**	**0.31**	**0.86**	0.239	1.30	0.84	2.02
Stage (III/IV)	**<0.001**		**0.27**	**0.16**	**0.45**	0.337	0.79	0.50	1.27
Residual disease after surgery (>2 cm)	**0.001**		**0.41**	**0.24**	**0.68**	0.886	0.97	0.63	1.49

**BOLD** P≤05 was considered statistically significant. Information for residual disease after surgery and response to therapy data are not available for Training Set.

### Methylation frequency of *VGF* and *PGP9.5* in the independent validation (IV) set of samples and clinicopathological correlation

**Figure 1:**
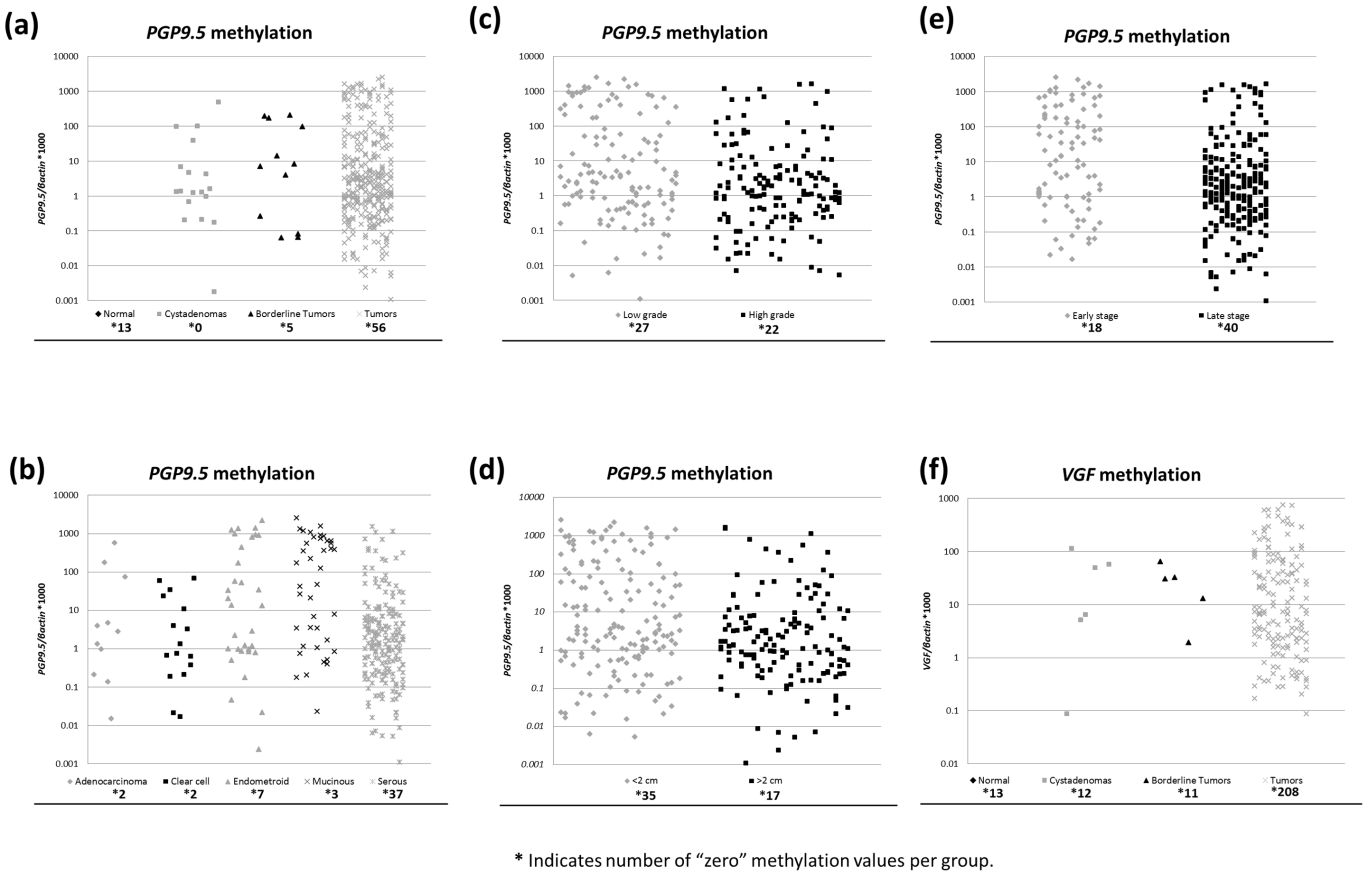
Representative scatter plots showing methylation levels of *PGP9.5* and *VGF* in ovarian tumor separated by samples' clincopathological characteristics. Calculation of the *PGP9.5* or *VGF* gene to *β-actin* ratios was based on the fluorescence emission intensity values for both the genes obtained by quantitative methylation-specific real-time PCR analysis. The obtained ratios were multiplied by 1,000 for easier tabulation. Zero values are indicated in the lower part of the graph, showing the amount of samples, as they cannot be plotted correctly on a log scale. (**A**) *PGP9.5* methylation values in normal samples (0/13, 0%), cystadenomas (12/17, 71%), borderline tumors (18/18, 100%) and ovarian tumors (316/372, 85%). (**B**) Methylation of *PGP9.5* throughout the histological types (O.R = 0.24, 95%C.I. [0.14–0.40], *p*<0.001). (**C**) *PGP9.5* methylation was significantly correlated with lower grade (O.R = 0.24, 95% C.I. [0.14–0.40], *p* = 0.012). (**D**) *PGP9.5* methylation was significantly correlated with absence of residual disease (lower than 2cm) (O.R = 0.41, 95%C.I. [0.24–0.68], *p* = 0.001). (**E**) *PGP9.5* methylation was significantly correlated with early stage of tumors (O.R = 0.26, 95%C.I.[0.16–0.45] *p*<0.001. (**E**) *VGF* methylation values and frequencies in normal samples (0/13, 0%), cystadenomas (5/16, 31%), borderline tumors (6/18, 33%) and ovarian tumors (158/366, 43%).

Based on the availability of samples and novelty of genes for OC and methylation frequencies in the training set, we selected 2 genes (*VGF* and *PGP9.5*) to test in an independent cohort of samples consisting of 372 carcinomas, 18 borderline tumors and 17 cystadenomas. Both *VGF* and *PGP9.5* methylation showed a cancer specific pattern, with 43% and 85% frequency respectively in tumors, while 0% in normals, as stated previously (**Table 2B**) (**Figure 1**). Interestingly high methylation frequency was observed for both genes in borderline tumors and cystadenoma (**Table 2B**) (**Figure 1**).

In the independent validation set, using univariate logistic regression analysis, we compared the methylation status of *VGF* and *PGP9.5* to demographic and clinicopathological parameters such as age, stage, histological type, grade, and presence of residual disease after surgery. *PGP9.5* methylation (using the cutoff above the 75th percentile of methylation ratio) was significantly correlated with lower grade and early stage of tumors (O.R = 0.52, 95% C.I. [0.31–0.86], *p* = 0.012) and (O.R = 0.27, 95% C.I.[0.16–0.45], *p*<0.001), respectively, as well as with absence of residual disease (O.R = 0.41, 95%C.I.[0.24–0.68], *p* = 0.001) and histological type (non-serous) (O.R = 0.24, 95%C.I.[0.14–0.40], *p*<0.001) (**Table 3B**) (**Figure 1**). A summary of methylation of *VGF* and *PGP9.5* with clinicopathological correlation in independent validation set are shown in **Table 3B**.

**Figure 2:**
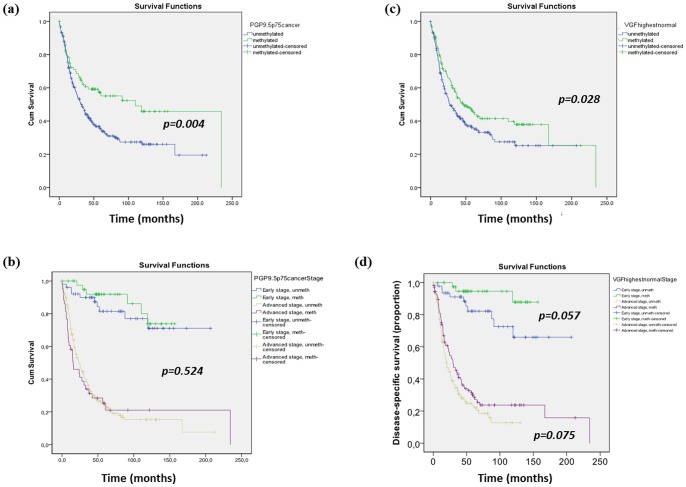
Kaplan-Meier curves for the studied populations stratified by gene methylation status and also stage. (**A**) Kaplan-Meier curve for the study population stratified for *PGP9.5* methylation. Overall survival was significantly higher in patients with *PGP9.5* methylation. By Cox regression univariate, the hazard ratio [HR] is 0.59, 95% CI [0.42–0.84], *p* = 0.004. (**B**) Kaplan-Meier curve for the study population stratified for *PGP9.5* methylation and stage. Patients with early stage (stages I and II) showed better survival than those with late stage (stages III and IV) (P<0.001). Both groups had similar survival when methylated *PGP9.5* was compared to unmethylated (*p* = 0.524). (**C**) Kaplan-Meier curve for the study population stratified for *VGF* methylation. Overall survival was significantly higher in patients with *VGF* methylation. By Cox regression univariate, the hazard ratio [HR] is 0.73 [95%CI; 0.55–0.97], *p* = 0.028. (**D**) Kaplan-Meier curve for the study population stratified for *VGF* methylation and stage. Patients with early stage (stages I and II) showed better disease specific survival than those with late stage (stages III and IV) (*p*<0.001). Both groups had a trend for better survival when *VGF* was methylated compared to unmethylated.

All the 372 cases of ovarian carcinoma of the independent validation set were available for follow up analysis. We used Cox Proportional Hazard Model to predict overall survival (OS) and disease specific survival (DSS) of these 372 patients associated with PH of *PGP9.5* and *VGF*. Median follow up for these patients were 30 months, ranging from 1 to 234 months. Consistent with previous observation, by a univariate cox regression analysis, we found correlation of poor survival with later stage (III/IV) of tumor (Hazard Ratio (HR) 8.22, 95% C.I [4.91–13.76], p<0.001), and presence of residual disease (HR 4.73, 95%CI [3.47–6.44], p<0.001) (**Table 4**). Methylation status of each of the genes was a critical factor for predicting patient OS and DSS. In univariate cox regression analysis, presence of *PGP9.5* methylation correlated significantly with better patient overall survival (HR 0.59, 95%CI [0.42–0.84], p = 0.004) and DSS (HR 0.57, 95%CI [0.39–0.82], p = 0.003). **Figure 2A** shows the Kaplan-Meier curve for OS depending on the methylation status of *PGP9.5*. However, these findings did not remain significant in the multivariate analysis (**Table 4**), we can observe a Kaplan-Meier curve for OS separated by stage depending on the methylation status of *PGP9.5* (**Figure 2B**). Similarly univariate cox regression and Kaplan Meier analysis (**Table 4**) were performed for *VGF*. OS was significantly higher in patients with *VGF* methylation (HR 0.73, 95% CI [0.55–0.97], p = 0.028) (**Figure 2C**), as well as DSS (HR 0.72, 95%CI [0.54–0.96], p = 0.027). After adjusting for tumor stage and presence of residual disease, in a multivariate model, *VGF* methylation remained as a good predictor of better survival: OS (HR 0.61, 95%CI [0.43–0.86], p<0.005) (**Table 4**) and DSS (HR 0.58, 95%CI [0.41–0.83], p<0.003). **Figure 2D** shows a Kaplan-Meier curve for DSS separated by stage depending on the methylation status of *VGF*.

**Table 4:** Cox proportional hazards model of variables predicting decreased overall survival

UNIVARIATE ANALYSIS				
Variables				
	P value	HR	95% C.I.	
Age (>61)	**0.001**	**1.57**	**1.19**	**2.06**
Histology (serous)	**<0.001**	**2.46**	**1.74**	**3.48**
Grade (III/undifferentiated)	**<0.001**	**1.91**	**1.42**	**2.56**
Stage (III/IV)	**<0.001**	**8.22**	**4.91**	**13.76**
Residual disease after surgery (>2 cm)	**<0.001**	**4.73**	**3.47**	**6.44**
*PGP9.5* methylated	**0.004**	**0.59**	**0.42**	**0.84**
*VGF* methylated	**0.028**	**0.73**	**0.55**	**0.97**
**MULTIVARIATE ANALYSIS**				
**Covariate**				
PGP9.5 methylated	0.524	1.15	0.74	1.80
Age (continuous)	0.378	1.01	0.99	1.02
Histology (serous)	0.671	1.10	0.70	1.73
Grade (III/undifferentiated)	0.769	1.05	0.75	1.48
Stage (III/IV)	**<0.001**	**4.56**	**2.26**	**9.22**
Residual disease after surgery (>2 cm)	**<0.001**	**2.70**	**1.84**	**3.97**
**Covariate**				
VGF methylated	**0.005**	**0.62**	**0.44**	**0.87**
Age (continuous)	0.215	1.01	1.00	1.02
Histology (serous)	0.509	1.16	0.74	1.81
Grade (III/undifferentiated)	0.529	1.12	0.79	1.58
Stage (III/IV)	**<0.001**	**4.04**	**2.02**	**8.11**
Residual disease after surgery (>2 cm)	**<0.001**	**2.76**	**1.88**	**4.04**
**Covariate**				
VGF methylated	**0.014**	**0.69**	**0.51**	**0.93**
Stage (III/IV)	**<0.001**	**4.67**	**2.67**	**8.16**
Residual disease after surgery (>2 cm)	**<0.001**	**2.79**	**2.00**	**3.88**

**BOLD** P≤.05 was considered statistically significant.

### *VGF* promoter methylation correlates with expression in cell lines and primary tumors; and overexpression of *VGF* inhibits OC cell growth *in vitro*

**Figure 3:**
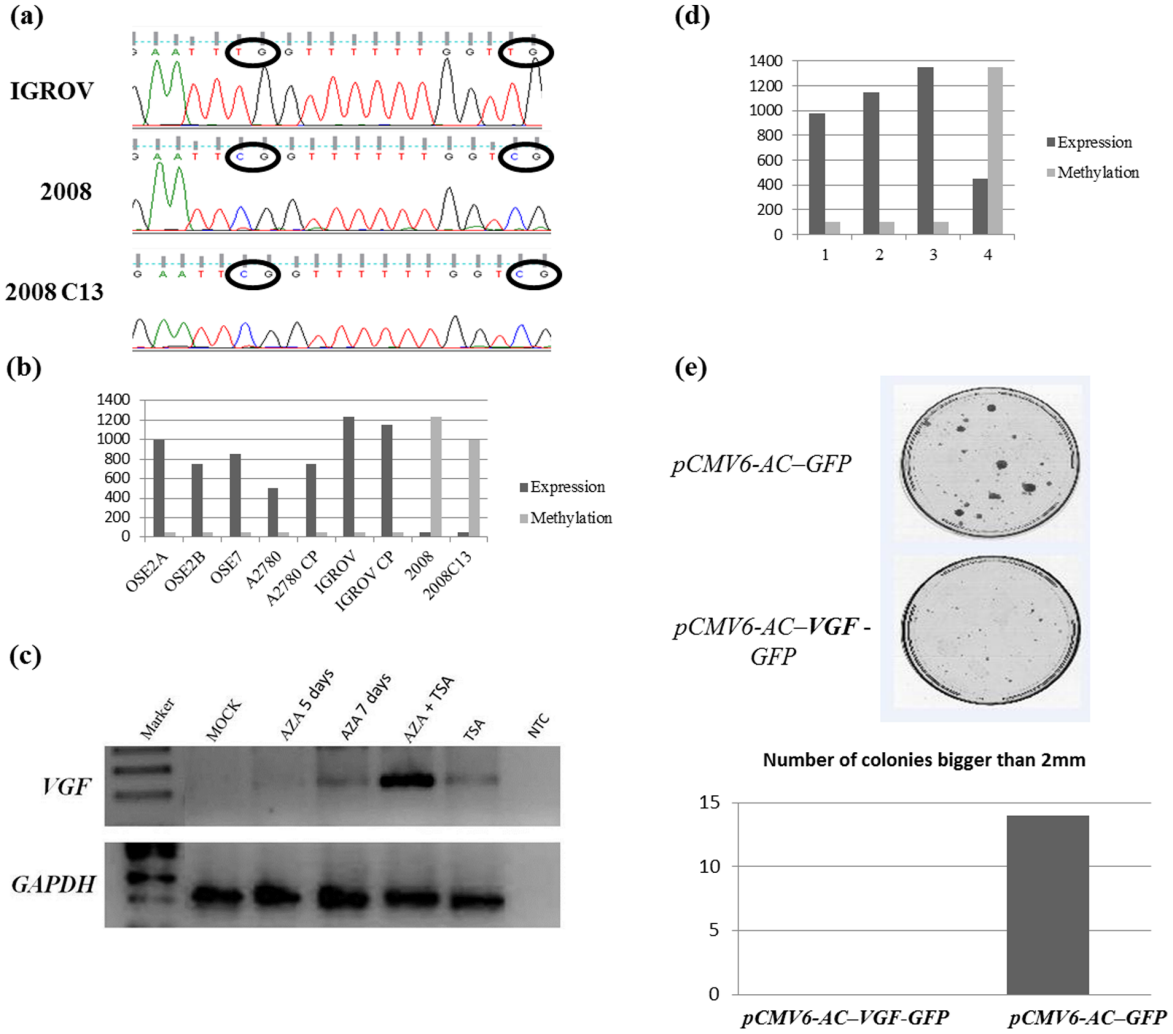
Analysis of *VGF* methylation and expression. (**A**) Three representative sequences (electropherograms) of promoter sequencing of *VGF* after Sodium bisulfite DNA conversion in OC cell lines. Upper panel shows IGROV with the respective unmethylated CG dinucleotides (we can observe the indicated TGs by a circle) and lower panel shows 2008 and 2008C13 with the respective CG dinucleotides methylated. All cytosines present after sodium bisulfite sequencing are corresponding to methyl cytosines. The thimidines represent the absence of methylation on the cytosines on that same spot. (**B**) Bar graph showing expression and methylation data side by side. Light grey bars represent methylation assessed by Quantitative Methylation Specific PCR (QMSP) in 6 tumor cell lines and 3 normal ovarian epithelium cells, Dark grey bars mRNA expression level by RT-PCR on the same cell lines. (**C**) 2008 ovarian cancer cell line treated with the demethylating agent 5-aza-2′-deoxycytidine (5-aza-dC) alone and in combination with the histone deacethylase inhibitor Trichostatin (TSA), or with TSA alone, and mock treated for all mentioned conditions. Re-expression of VGF was determined after 5 days, 7 days of treatment, treatment with TSA alone or the combination of both drugs. AZA, 5-aza-dC; NTC, non template control (water). (**D**) Bar graph showing expression and methylation data side by side. Light grey bars represent methylation assessed by QMSP in 4 tumors, Dark grey bars mRNA expression level by RT-PCR on the same tumors. (**E**) Ectopic expression of *VGF* inhibits tumor cell growth. Upper panel: The effect of ectopic VGF-expression on ovarian carcinoma cell clonogenicity was investigated by monolayer colony formation assay. Cells were transfected with *VGF* overexpression vector (*pCMV6-AC–* ***VGF***–*GFP*) or control vector (*pCMV6-AC*-*GFP*), and selected with G418 on the ovarian cancer cell line 2008 C13. Lower panel, Bar graph showing the number of colonies observed (larger than 2mm). No colonies were observed after over expressing VGF containing vector while numerous colonies were observed after control vector transfection.

We tested OC derived cell lines (A2780 and A2780CP, 2008 and 2008C13, and IGROV and IGROV CP) as well as normal ovarian epithelium cell lines (OSE2A, OSE2B and OSE7) for *VGF* methylation. **Figure 3A** shows the representative bisulfite sequencing chromatogram for 2008, 2008C13 and IGROV OC cell lines. We further tested the methylation status of *VGF* for all the latter cell lines by QMSP. **Figure 3B** shows normalized methylation values of these cell lines. As evident in the **Figure 3** ovarian normal and 4 OC cell lines do not have any promoter methylation for *VGF* while 2 ovarian cell lines (2008 and 2008C13) demonstrated PH (**Figure 3B**). To determine whether promoter methylation of VGF has any effect on its expression, we assessed mRNA expression level of *VGF* by Quantitative RT-PCR of the same cell lines and indeed observe that promoter methylation inversely correlated with *VGF* expression in all cell lines (**Figure 3B**). No expression of *VGF* in 2008 and 2008C13 OC cell lines were observed with promoter methylation. To further confirm methylation indeed related to expression, we treated the 2008 OC cell line with the demethylating agent 5-aza-2′-deoxycytidine (5-aza-dC) alone and in combination with the histone deacethylase inhibitor Trichostatin (TSA), or with TSA alone, and mock treated for all mentioned conditions. Re-expression of *VGF* was determined after 5 and more noticeable 7 days of treatment, treatment with TSA alone or the combination of both drugs (**Figure 3C**). Notably, synergistic treatment of 5-aza-dC and TSA induced robust expression of *VGF*.

To determine the correlation of *VGF* PH with gene expression in *in vivo* situations, we assessed the methylation status of *VGF* and mRNA level expression in 4 OC patients (matched samples). We observed expression of *VGF* generally correlated with promoter methylation (**Figure 3D**). The lack of direct relationship between the presence of methylation and total absence of expression can be due to the fact that our samples still retain normal cells that could be responsible for this low level expression. Another possible reason could be the level of methylation observed may not be enough to completely shut down *VGF* expression.

To determine the biologic consequences of VGF, we forcefully introduced *VGF* into two OC cell lines (2008 and 2008C13) and performed focus formation assays. *VGF* expression vector (*pCMV6-AC–VGF*–*GFP,* OriGene Technologies, Inc., Rockville, MD), as well as the empty vector (*pCMV6-AC*-*GFP*, OriGene Technologies, Inc.) were transfected into 2008 and 2008C13 OC cell lines that have fully methylated and silenced *VGF*. The colony focus formed by *VGF*-transfected cells were significantly less and smaller in size than those of empty vector transfected cells. Representative data are shown in **Figure 3E**.

## Discussion

Our main goal in this study was to define a set of methylation markers capable of distinguishing ovarian carcinoma and normal ovarian tissue, and also to identify a panel of biomarkers that are correlated with patient's clinicopathological characteristics.

We selected the initial 13 genes to be analyzed based on previous reports in the literature. Aberrant methylation of *GSTP1* and *MGMT* were exclusively demonstrated in invasive ovarian carcinomas [20], differentiating between ovarian tumors with low malignant potential and invasive ovarian tumors. *GSTP1* and *MGMT*, (involved in DNA repair/drug detoxification) methylation have also been associated with a higher response to chemotherapy in OC [21]. The role of *ESR1* in OC remains unclear; some investigators have found hypermethylation in malignant ovarian tumors and low malignant potential ovarian tumors [22]. *KIF1A* is an anterograde motor protein that transports membranous organelles along axonal microtubules. As a part of “Cancer Methylome” discovery, we tested a small number of OC samples and found a high frequency of PH of *KIF1A* in OC [17]. Cancer-specific methylation of activated kinase 3 (*PAK3*) was found in several neoplasms, including OC with high frequency [17]. *HIC1* is a potential TSG and found to be methylated in several solid tumors including OC [23]–[25]. In the study by Tokumaru et al. [26] *PGP9.5* was found to have a high frequency of PH in a large panel of primary tumors, and their data support the notion that this gene is a TSG that is inactivated by PH or gene deletion in several types of human cancers. Some genes were described as hypermethylated in other cancer types, these include *SSBP2*, [27], *CCNA1* [28], [29], *AIM1* [30], *VGF* [17], *FKBP4* [31], and we decided to evaluate their promoter methylation status in ovarian tissues samples.

From the initial 13 genes analyzed in the training set, two genes were further explored in an independent set of samples. *ESR1*, *PGP9.5*, *HIC1* and *VGF* showed cancer specific methylation pattern in the training set explored, and we chose *PGP9.5* and *VGF* to be further explored in an independent validation set. Multiple groups had previously shown *HIC1* to be frequently methylated in OC samples [23]–[25] and our findings could corroborate these data, although the reported frequencies vary from 17 to 50%, all using less sensitive techniques than ours that showed a frequency of 65%. *ESR1* has been found hypermethylated in malignant ovarian tumors and low malignant potential ovarian tumors [22], but was the least frequent in our training set (**Table 2**). So due to lack of novelty and sample availability, we did not included *HIC1* and *ESR1* in the validation set.

*PGP9.5*, also known as *UCHL1*, is a neuro-specific peptide that removes ubiquitin from ubiquinated proteins and prevents them from targeted degradation by proteasomes [32]. Our group and others showed *PGP9.5* inactivation by PH in several types of solid tumors [28], [33], [34]. Mizukami et al. observed a frequent methylation pattern of PGP9.5 in colorectal cancer where it was more frequently methylated in patients with earlier stages of colorectal cancer than in metastatic cases [35]. We are reporting for the first time that *PGP9.5* is significantly more methylated in early stage and lower grade of OC. There is controversy regarding the role of *PGP9.5* as a TSG or oncogene in cancer. In the study by Tokumaru et al. *PGP9.5* was found to have a high frequency of PH in a large panel of primary tumors, and their data support the notion that this gene is a TSG that is inactivated by PH [26]. On the other hand, there are numerous reports that PGP9.5 is over-expressed in a subset of primary cancers [26], [36]. Over-expression in primary cancer tissues could be the cause of or the result of transformation. If it is the cause of transformation, PGP9.5 would be an oncogenic molecule, but the clinical profile reported here does not support this notion for OC as we observed significantly more *PGP9.5* methylation in early stage OC and no methylation was determined in normal ovarian epithelium. In OC, *PGP9.5* has been analyzed before by conventional MSP and bisulfite sequencing, and demonstrated a very low frequency in the 17 tumors analyzed (6%) [37]. The primers used in that study are different from the primers we used in this study. In addition Quantitative MSP is thought to be at least ten times more sensitive than the conventional MSP they used in their study. Addition of probe within the PCR product in QMSP provides additional specificity by QMSP assay than conventional MSP.

VGF is a secreted neuropeptide, recently reported to be methylated in cancer in a “Cancer Methylome” discovery approach by our group [17]. We found high methylation frequency of *VGF* in OC. We recently reported cancer specific methylation of *VGF* in two other hormone related cancers (breast and testicular) [31], [38]. To our knowledge, there are no further reports linking VGF and cancer. It will be interesting to further explore the biologic relevance of VGF for the development of hormone related cancer. VGF has been shown to play an essential role in body weight, basal metabolism and nutrition [39]. In our study, PH of *VGF* correlated with loss of gene expression in cancer cell lines and primary ovarian tumor, and re-expression of *VGF* could be obtained by inhibiting DNA methylation with 5-aza-dC. However 5-aza-dC may be non-specific and induce *VGF* expression may be due to alterations of related signaling pathways and demethylating other genes which regulate the expression of *VGF*. While additional studies are necessary to accurately understand the role of *VGF* gene silencing during the multistep process of tumorigenesis, our results argue that *VGF* may be one of the most frequent targets of unscheduled silencing induced by aberrant PH in the genesis of OC. It is well established that DNA methylation of the CpG island in the promoter region is causally involved in gene silencing [17], therefore a tight correlation between *VGF* CpG island hypermethylation in cancer cell lines and loss of gene expression in these cells provides an explanation for the loss or inactivation of *VGF* previously reported in different pathological conditions [40]. Interestingly we found synergistic effect of 5-aza-dC and TSA for the induction of VGF expression. So studies on histone modifications on VGF may also be interesting. *VGF* knockout mice are reported to be hyperactive and hypermetabolic [41] and tumors are also known to be hypermetabolic. Further studies could be extended in tumors to analyze this relationship in *in vitro* and *in vivo* model systems. From a clinical point of view, our findings suggested that 5-aza-dC treatment is able to restore *VGF* expression in cancer cell lines and that over-expression of exogenous *VGF* inhibit colony formation of OC cells. So DNA hypermethylation of *VGF* may represent an attractive target for intervention strategies. However, further biological roles of *VGF* in tumorigenesis need to be elucidated.

From the outcome perspective, the correlation of presence of PH of these two genes (*VGF* and *PGP9.5*) with better overall and disease specific survival is a potential indication of its utility in clinics. The presence of methylation seems to be contributing for the occurrence of cancer but, in this case, conferring better prognosis. Inconsistent activity in developing tumorigenesis were reported for PGP9.5 [26]. There are very limited functional studies that have been performed for VGF and, to our knowledge, no functional studies has been reported for *VGF* in cancer. Although it needs to be proved, hypothetically one can argue that these genes may have diverse roles in cancer. An example to support this argument is *MGMT* promoter methylation; while promoter methylation of *MGMT* has been reported as frequent in several cancer types, thus being correlated with cancer initiation and/or progression; Glioma patients harboring methylation of *MGMT* were found to be more responsive to alkylating agents than those without this epigenetic alteration [42], demonstrating its potential use in clinics. Our data showed significance in univariate analysis but not in multivariate analysis for *PGP9.5*. However, considering that methylation of *PGP9.5* was positively correlated with the presence of residual disease and early stage, which are strong prognostic factors for OC in univariate disease, it is understandable why *PGP9.5* methylation does not represent an independent survival predictor when used in conjunction with this clinical parameters. Interestingly, we found that *VGF* methylation is an independent factor for predicting better survival for OC. The clinical usefulness of an independent prognostic factor is its ability to more accurately predict patient survival when used with other known prognostic factors. Such a marker would be invaluable to physicians and patients in selecting treatment options. Among the known predictors for OC, higher stages and presence of residual diseases after surgery are the two most critical poor survival predictors with a HR of 8.22 and 4.73 respectively in our independent validation set of samples. When we performed multivariate analysis of *VGF* with these two factors, *VGF* methylation was still significantly related to better overall survival. To explore the utility of *VGF* methylation as a clinical tool for predicting survival of OC patients at diagnosis, a higher sample size with longer follow-up and well annotated clinical data is needed. Although biological relevance data is not available for methylation of *PGP9.5* and *VGF* in OC, our findings of methylation based good prognosis markers are supported by a recent study that reported PH of potential TSG *FBXW7/hCDC4-*β being related to favorable prognosis of primary breast cancer [43].

Several methylation studies have been reported previously in OC, and some of cancer specific methylated genes were shown to be correlated with stage, prognosis, survival and resistance to therapy. For example, *IGFBP-3* hypermethylation was associated with disease progression and death in OC, particularly in patients with early stage disease [44]. A set of markers found to be correlated with early stage in serum or plasma samples of OC patients was composed of *RASSF1A*, *BRCA1, APC, CDKN2A* and *DAPK* [4]. From these, *RASSF1A,* has been shown in another study to bind to tubulin and stabilize microtubules, thereby, assisting paclitaxel in mediating the prevention of spindle assembly [45]. In addition, silencing of *hMLH1* has been linked with resistance to platinum drugs as well [46]. Ozdemir et al. [47] analyzed 24 genes by two different techniques that showed identical results and observed *CDKN2B* as frequently methylated in OC and observed that 40% of the samples analyzed had at least one of these genes methylated. Other genes like *SFRP1, SFRP2, SOX1, LMX1A, TUSC3* and *HOXA11* have been found to be correlated with recurrence and overall survival/outcome of OC patients, being *HOXA11* independently associated with poor outcome [48]–[50]. Hypermethylation of *TUSC3*, a putative TSG, was associated with significant shorter progression-free and overall survival rates in OC, independently of other known risk factor [50]. Dai et al. showed that *NLD1* and *DVL1* methylation are independently associated with progression free survival, being potential markers of good prognosis [51]. Zeller et al. [52] examined genome-wide methylation in order to establish differences and similarities among ovarian tumors classified as Low-grade serous carcinomas (LGSC), serous borderline ovarian tumors (SBOT) and benign serous tumors (BST), since few is known about the progression from benign lesions of the ovary to serous carcinomas, as well as what are the characteristics that allow the genesis of LGSC from borderline tumors. They found that DNA methylation profiles could separate between BST and LGSC, but not between LGSC and SBOT. They could not identify a classifier for distinguishing SBOT with benign or malignant like methylation profile, however they could observe that LGSC and SBOT have different profiles than benign lesions, being very similar among each other. All these markers represent some examples of how epigenetic changes, like DNA methylation, can drive genes to have a role in ovarian carcinogenesis. More examples of markers with a role in OC can be found in a more extensive and detailed review from our group [53].

It has been long recognized that inactivation by DNA PH is one of the hallmarks of cancer. We chose to use a candidate gene approach, where genes known to be inactivated in several types of cancer were examined in ovarian samples. In this work, we made the option of examining independent cohorts of OC samples to truly elucidate the PH frequency and importance of our selected candidate genes. The present study not only contributes with an analysis of a panel of methylation regulated genes in ovarian carcinoma, revealing genes with cancer specific patterns, but also highlights the enormous possibilities of identifying new molecular markers that will allow the translation of this knowledge to the daily clinics, permitting the accurate selection of patients to different therapies based on their molecular profile.

## Supporting Information

File S1 **Supporting Information contain Table S1 and Table S2. Table S1.** Genes selected and their proposed function. **Table S2.** Primers and probes sequences and annealing temperatures (T°C) used for QMSP.
(DOCX)
