# Correction: Association of Promoter Methylation of *VGF* and *PGP9.5* with Ovarian Cancer Progression

**DOI:** 10.1371/annotation/ccd51e37-fdf3-466e-b79f-2c390df9ab28

**Published:** 2013-10-29

**Authors:** Mariana Brait, Leonel Maldonado, Maartje G. Noordhuis, Shahnaz Begum, Myriam Loyo, Maria Luana Poeta, Alvaro Barbosa, Vito M. Fazio, Roberto Angioli, Carla Rabitti, Luigi Marchionni, Pauline de Graeff, Ate G. J. van der Zee, G. Bea A. Wisman, David Sidransky, Mohammad O. Hoque

The name of the third author is: Maartje G. Noordhuis

The correct version of the citation is: Brait M, Maldonado L, Noordhuis M, Begum S, Loyo M, et al. (2013) Association of Promoter Methylation of *VGF* and *PGP9.5* with Ovarian Cancer Progression. PLoS ONE 8(9): e70878. doi:10.1371/journal.pone.0070878 

