# Supplementary material for: Association of Promoter Methylation of VGF and PGP9.5 with Ovarian Cancer Progression
Source: PLoS One. 2013 Sep 27;8(9):e70878. doi: 10.1371/journal.pone.0070878 (PMC3785492; doi:10.1371/journal.pone.0070878)
Supplement: File S1 — Supporting Information contain Table S1 and Table S2. Table S1. Genes selected and their proposed function. Table S2. Primers and probes sequences and annealing temperatures (T°C) used for QMSP. (DOCX) [file pone.0070878.s001.docx]

**Supplemental Table S1**

|  |  | |  | |  | |
| --- | --- | --- | --- | --- | --- | --- |
| **Supplemental Table S1. Genes selected and their proposed function** | | | | | | |
| **Gene** | | **Chromosomal location** | | **Name** | | **Proposed Function** |
| *AIM1* | | 6q21 | | Absent in melanoma 1 | | Unknown |
| *APC* | | 5q21-q22 | | Adenomatous polyposis coli | | This gene encodes a tumor suppressor protein that acts as an antagonist of the Wnt signaling pathway. It is also involved in other processes including cell migration and adhesion, transcriptional activation, and apoptosis. |
| *CCNA1* | | 13q12.3-q13 | | Cyclin A1 | | The protein encoded by this gene belongs to the highly conserved cyclin family, whose members are characterized by a dramatic periodicity in protein abundance through the cell cycle. |
| *ESR1* | | 6q25.1 | | Estrogen receptor 1 | | This gene encodes an estrogen receptor, a ligand-activated transcription factor composed of several domains important for hormone binding, DNA binding, and activation of transcription. |
| *FKBP4* | | 12p13.33 | | FK506 binding protein 4 | | peptidylprolyl cis-trans isomerase, protein binding, protein folding |
| *GSTP1* | | 11q13 | | Glutathione S-transferase pi 1 | | DNA repair/drug detoxification |
| *HIC1* | | 17p13.3 | | Hypermethylated in cancer 1 | | Zinc finger transcription factor; potential tumor suppressor |
| *KIF1A* | | 2q37.3 | | Kinesin family member 1A | | Anterograde motor protein that transports membranous organelles along axonal microtubules. |
| *MGMT* | | 10q26 | | O-6-methylguanine-DNA methyltransferase | | DNA repair/drug detoxification |
| *PAK3* | | Xq22.3-q23 | | Activated kinase 3 | | PAK proteins are critical effectors that link Rho GTPases to cytoskeleton reorganization and nuclear signaling. |
| *PGP9.5* | | 4p14 | | Ubiquitin carboxyl-terminal esterase L1 | | Member of a gene family whose products hydrolyze small C-terminal adducts of ubiquitin to generate the ubiquitin monomer. |
| *SSBP2* | | 5q14.1 | | ssDNA-binding protein2 | | DNA binding |
| *VGF* | | 7q22 | | VGF nerve growth factor inducible precursor | | Neuropeptide |

**Supplemental Table S2**

| **Supplemental Table S2.** Primers and probes sequences and annealing temperatures (T^o^C) used for QMSP. | | | | |
| --- | --- | --- | --- | --- |
| **Gene** | **Forward 5´-3´ (primer)** | **Probe 5´-3´ (6-FAM-5’- 3’-6-TAMRA)** | **Reverse 5´-3´ (primer)** | **T^o^C** |
| *βActin* | TGGTGATGGAGGAGGTTTAGTAAGT | ACCACCACCCAACACACAATAACAAACACA | AACCAATAAAACCTACTCCTCCCTTAA | 60 |
| *AIM1* | CGCGGGTATTGGATGTTAGT | GGGAGCGTTGCGGATTATTCGTAG | CCGACCCACCTATACGAAAA | 60 |
| *APC* | GAACCAAAACGCTCCCCAT | CCCGTCGAAAACCCGCCGATTA | TTATATGTCGGTTACGTGCGTTTATAT | 60 |
| *CCNA1* | TCGCGGCGAGTTTATTCG | CGTTATGGCGATGCGGTTTCGG | CCGACCGCGACAAACG | 60 |
| *ESR1* | GGCGTTCGTTTTGGGATTG | CGATAAAACCGAACGACCCGACGA | GCCGACACGCGAACTCTAA | 60 |
| *FKBP4* | GTTCGTGGTGACGGTCGGTTTCGGG | CAAACTACGAAATAACAATAACGACGC | ATCCGCTACGCCTACGACG | 58 |
| *GSTP1* | AGTTGCGCGGCGATTTC | CGGTCGACGTTCGGGGTGTAGCG | GCCCCAATACTAAATCACGACG | 60 |
| *HIC1* | GTTAGGCGGTTAGGGCGTC | CAACATCGTCTACCCAACACACTCTCCTACG | CCGGGCGCCTCCATCGTGT | 60 |
| *KIF1A* | GCGCGATAAATTAGTTGGCGATT | CCTCCCGAAACGCTAATTAACTACGCG | CTCGACGACTACTCTACGCTAT | 58 |
| *MGMT* | CGAATATACTAAAACAACCCGCG | AATCCTCGCGATACGCACCGTTTACG | GTATTTTTTCGGGAGCGAGGC | 60 |
| *PAK3* | TTACGGTCGTCGTTATTATCG | AACCAAAAAAAATAAAAAATCACAACCG | ACCGAAAATTCTACCCTTCG | 60 |
| *PGP9.5* | CGGCGAGTGAGATTGTAAGGTT | TTCGGTCGTATTATTTCGCGTTGCGTAC | GAACGATCGCGACCAAATAAATAC | 60 |
| *SSBP2* | ATTTTTGCGGTCGTAGCGGT | ATATCCAAAACGCCGCGAAACTCC | TTCTACGACAAATCTAACGAA | 60 |
| *VGF* | GGATAGCGTTCGTAGGCG | GCGCCCAAAAACGACGTAAACCTAAATAC | AAAAACCGAATTCCCCACCCCG | 60 |
